# Accuracy, Reproducibility, and Responsiveness to Treatment of Home Spirometry in Cystic Fibrosis: Multicenter, Retrospective, Observational Study

**DOI:** 10.2196/60892

**Published:** 2024-12-03

**Authors:** Martinus C Oppelaar, Hanneke AC van Helvoort, Michiel AGE Bannier, Monique HE Reijers, Hester van der Vaart, Renske van der Meer, Josje Altenburg, Lennart Conemans, Bart L Rottier, Marianne Nuijsink, Lara S van den Wijngaart, Peter JFM Merkus, Jolt Roukema

**Affiliations:** 1 Department of Pediatric Pulmonology Amalia Children's Hospital Radboud University Medical Center Nijmegen Netherlands; 2 Department of Pulmonary Diseases Radboud University Medical Center Nijmegen Netherlands; 3 Department of Paediatric Pulmonology MosaKids Children's Hospital Maastricht University Medical Centre+ Maastricht Netherlands; 4 Department of Pulmonary Diseases University Medical Center Groningen University of Groningen Groningen Netherlands; 5 Department of Pulmonology Haga Teaching Hospital The Hague Netherlands; 6 Department of Respiratory Medicine Amsterdam University Medical Centers University of Amsterdam Amsterdam Netherlands; 7 Department of Respiratory Medicine Maastricht University Medical Centre+ Maastricht Netherlands; 8 Division of Respiratory & Age-related Health Department of Respiratory Medicine NUTRIM Institute of Nutrition and Translational Research in Metabolism Maastricht Netherlands; 9 Department of Pediatric Pulmonology and Pediatric Allergology Beatrix Children's Hospital University Medical Center Groningen, University of Groningen Groningen Netherlands; 10 Groningen Research Institute for Asthma and COPD University Medical Center Groningen University of Groningen Groningen Netherlands; 11 Juliana Children's Hospital Haga Teaching Hospital The Hague Netherlands

**Keywords:** telemonitoring, digital health, telespirometry, remote monitoring, cystic fibrosis, pediatrics, reliability, mobile phone, hereditary, chronic pulmonary inflammation, pulmonary infections, morbidity, mortality, chronic respiratory disease

## Abstract

**Background:**

Portable spirometers are increasingly used to measure lung function at home, but doubts about the accuracy of these devices persist. These doubts stand in the way of the digital transition of chronic respiratory disease care, hence there is a need to address the accuracy of home spirometry in routine care across multiple settings and ages.

**Objective:**

This study aimed to assess the accuracy, reproducibility, and responsiveness to the treatment of home spirometry in long-term pediatric and adult cystic fibrosis care.

**Methods:**

This retrospective observational study was carried out in 5 Dutch cystic fibrosis centers. Home spirometry outcomes (forced expiratory volume in one second [FEV_1_], and forced vital capacity [FVC]) for 601 anonymized users were collected during 3 years. For 81 users, data on clinic spirometry and elexacaftor/tezacaftor/ivacaftor (ETI) use were available. Accuracy was assessed using Bland-Altman plots for paired clinic-home measurements on the same day and within 7 days of each other (nearest neighbor). Intratest reproducibility was assessed using the American Thoracic Society/European Respiratory Society repeatability criteria, the coefficient of variation, and spirometry quality grades. Responsiveness was measured by the percentage change in home spirometry outcomes after the start of ETI.

**Results:**

Bland-Altman analysis was performed for 86 same-day clinic-home spirometry pairs and for 263 nearest neighbor clinic-home spirometry pairs (n=81). For both sets and for both FEV_1_ and FVC, no heteroscedasticity was present and hence the mean bias was expressed as an absolute value. Overall, home spirometry was significantly lower than clinic spirometry (mean ΔFEV_1clinic-home_ 0.13 L, 95% CI 0.10 to 0.19; mean ΔFVC_clinic-home_ 0.20 L, 95% CI 0.14 to 0.25) and remained lower than clinic spirometry independent of age and experience. One-way ANOVA with post hoc comparisons showed significantly lower differences in clinic-home spirometry in adults than in children (Δmean 0.11, 95% CI –0.20 to –0.01) and teenagers (Δmean 0.14, 95% CI –0.25 to –0.02). For reproducibility analyses, 2669 unique measurement days of 311 individuals were included. Overall, 87.3% (2331/2669) of FEV_1_ measurements and 74.3% (1985/2669) of FVC measurements met reproducibility criteria. Kruskal-Wallis with pairwise comparison demonstrated that for both FVC and FEV_1_, coefficient of variation was significantly lower in adults than in children and teenagers. A total of 5104 unique home measurements were graded. Grade E was given to 2435 tests as only one home measurement was performed. Of the remaining 2669 tests, 43.8% (1168/2669) and 43.6% (1163/2669) received grade A and B, respectively. The median percentage change in FEV_1_ from baseline after initiation of ETI was 19.2% after 7-14 days and remained stable thereafter (n=33).

**Conclusions:**

Home spirometry is feasible but not equal to clinic spirometry. Home spirometry can confirm whether lung functions remain stable, but the context of measurement and personal trends are more relevant than absolute outcomes.

## Introduction

Cystic fibrosis (CF) is an autosomal recessive hereditary condition caused by a range of genetic defects in the gene coding for the cystic fibrosis transmembrane conductance regulator (CFTR) protein [1]. The defect leads to increased sputum viscosity which causes chronic pulmonary inflammation and predisposes to recurrent pulmonary infections. Early detection and treatment of pulmonary deterioration is imperative for people with CF to prevent morbidity and mortality [1,2]. The forced expiratory volume in one second (FEV_1_) measured by spirometry is the most reliable parameter for early detection of this pulmonary deterioration [1,2].

Traditionally, spirometry is performed in a clinic guided by trained pulmonary function technicians, but small portable spirometers are also increasingly used by people with CF at home. Many of these portable spirometers have been validated and cleared by regulatory bodies such as the Federal Drug Agency and European Union authorities. However, both the unsupervised setting as well as the quality of these devices cause persistent doubts about the reliability of home spirometry among both health care professionals and people with CF [3]. These doubts stand in the way of the digital transition of CF care as well as of the wider field of chronic respiratory disease care.

Several recent studies show that home spirometry is consistently lower than clinic spirometry and has more intertest variability during stable periods [4-8]. In contrast, other studies argue that unsupervised home spirometry can yield similarly acceptable and reliable results as clinic spirometry [9-11]. Importantly, accuracy has almost exclusively been studied in prospective studies or within trials that are not reflective of real-life clinical practice. This might have introduced selection bias of skilled and motivated participants, and these studies might have missed changes in accuracy as home spirometry becomes repetitive for users over time.

This study aims to assess the accuracy, reproducibility, and responsiveness to treatment of home spirometry in long-term regular CF care in 5 Dutch CF centers between April 2020 and December 2022.

## Methods

### Study Design

This was a retrospective, multicenter, observational study on accuracy, reproducibility, and responsiveness to treatment of home spirometry in regular CF care performed in 5 Dutch CF centers (Radboud University Medical Center, Nijmegen; University Medical Center Groningen; Maastricht University Medical Center; Amsterdam University Medical Center; and Haga Hospital, The Hague).

We combined a large dataset of anonymous home spirometry outcomes dataset 1 (DS1) with a smaller dataset of clinical outcomes from participants in a remote monitoring study dataset 2 (DS2). DS1 consisted of all home spirometry outcomes of all users of the program (601 people with CF) from April 2020 until December 2022. DS2 consisted of clinical outcomes, including clinic spirometry, of 81 people with CF spread across the 5 participating centers who provided informed consent for the collection of their clinical data. Participants within DS2 could be coupled to their home spirometry outcomes in DS1 for accuracy analyses. Textbox 1 provides a summary of which datasets are used in separate analyses.

**Summary of datasets used and total eligible individuals per analysis**

- Accuracy: Home spirometry measurements from dataset 1 combined with clinic spirometry measurements from dataset 2. Total eligible participants: n=81.
- Reproducibility and quality grading: All same-day home spirometry measurements from dataset 1. Total eligible participants: n=601
- Responsiveness: Home spirometry measurements from dataset 1 combined with individual elexacaftor/tezacaftor/ivacaftor start dates from dataset 2. Total eligible participants: n=81.

### Portable Spirometer

The CE-marked Spirobank Smart portable spirometer (Medical International Research, MIR) is used in our population. The spirometer is connected to a dedicated smartphone app (Android, iOS). Spirometers were donated by the Dutch Cystic Fibrosis Foundation. Details and screenshots of the remote monitoring program (RMP) and spirometry module can be found in a previous publication [3]. The spirometers capture FEV_1_, forced vital capacity (FVC), peak expiratory flow (PEF), FEV_1_-to-FVC ratio, and forced expiratory flow at 25%-75%. The spirometer uses an algorithm that only allows submission of lung function tests that meet the 2005 American Thoracic Society/European Respiratory Society (ATS/ERS) spirometry criteria (volume max 10 L; volume accuracy ±3% or 0.05 L; flow range 960 L/minute; flow accuracy ±5% or 10.2 L/minute) [12,13].

CF centers carried responsibility for the implementation of the RMP and portable spirometers within their own needs and capacities [3]. In general, people with CF who were deemed able to reliably perform spirometry at home were eligible to receive a portable spirometer. People with CF were trained to use the device in the clinic, but home measurements itself were unsupervised. People with CF were generally instructed to perform 3 acceptable home spirometry maneuvers during every lung function measurement. The frequency of lung function measurements differed between hospitals and measurements were mostly patient-initiated, hence home spirometry was often used on indication (eg, during changing symptoms) [3].

### Statistical Analyses

All statistical analyses were performed in SPSS Statistics (version 27; IBM Corp). Statistical methods are described per study objective.

### Accuracy

For accuracy analyses, we paired clinic spirometry measurements from DS2 with home spirometry measurements from DS1 for participants in both datasets. Accuracy was assessed for home and clinic spirometry outcomes on the same day and with the nearest neighbor (NN) approach (home spirometry was compared with clinic spirometry performed within a maximum of 7 days from each other) [5]. For both pairs, Bland-Altman plots with 95% limits of agreement were used for FEV_1_, FVC, PEF, and FEV_1_/FVC ratio [14]. Differences between home and clinic FEV_1_ (ΔFEV_1clinic-home_) and FVC (ΔFVC_clinic-home_) were considered clinically significant when they exceeded 150mL as defined by the limit of acceptable repeatability in the ATS/ERS spirometry consensus statement [15].

One-way ANOVA was used to compare ΔFEV_1clinic-home_ and ΔFVC_clinic-home_ between age groups (6-12 years, 12-18 years, >18 years). ΔFEV_1clinic-home_ and ΔFVC_clinic-home_ were also plotted by age groups, by the time of use in days, and by the percentage of individual participants’ contribution to the overall amount of paired measurements (ie, less than 1 percent, between 1 and 3 percent, more than 3 percent).

### Reproducibility

All anonymous home spirometry outcomes from DS1 were used to assess the reproducibility of same-day home spirometry measurements (intratest reproducibility). For measurements with 2 or more home spirometry maneuvers, the 2 to 3 best FEV_1_ or FVC maneuvers were assessed separately. Reproducibility was assessed using repeatability criteria of the ATS/ERS consensus statement for FEV_1_ and FVC which states that measurements are repeatable when the difference between the largest and second-largest FEV_1_ or FVC is smaller or equal to 0.150 L [15]. We also calculated the coefficient of variation (CV; intratest SD/intratest mean 100). CV between age categories was analyzed using the Kruskal-Wallis test. To examine reproducibility over time, the reproducibility of home spirometry was also assessed in 3 subgroups: measurements performed between 0-100 days after initiation, between 300-400 days after initiation, and between 600-700 days after initiation.

### Quality Grading

All home spirometry tests in DS1 were graded according to the ATS/ERS technical statement [15,16]. In short, grade A includes ≥3 acceptable tests within 0.150 L; grade B includes 2 acceptable tests within 0.150 L; grade C includes ≥2 acceptable tests within 0.200 L; grade D includes ≥2 acceptable tests within 0.250 L; and grade E includes ≥2 acceptable tests within more than 0.250 L or one acceptable test [15,16]. Grading criteria were applied to the highest FEV_1_ and FVC results separately. Grade F (ie, no acceptable tests) tests could not be assessed as the Spirobank Smart only allows submission of maneuvers that meet ATS/ERS quality criteria.

### Responsiveness to Treatment

Individual start dates of a new CFTR modulator drug elexacaftor/tezacaftor/ivacaftor (ETI) were available for participants in DS2. The absolute percentage change from baseline in home spirometry outcomes in DS1 of these participants was analyzed. Baseline FEV_1_ or FVC was defined as the average FEV_1_ or FVC of all home spirometry outcomes during the 2 months before the start of ETI. Absolute percentage change in FEV_1_ and FVC from baseline were then analyzed during multiple time periods after initiation of ETI: 0-7 days after start, 7-14 days after start, 14-21 days after start, 21-28 days after start, and 28 days to 2 months after start.

### Ethical Considerations

This study was part of a larger study of remote monitoring in regular CF care for which approval of the Medical Research Involving Human Subjects Act was waived by the local ethical committees [3] (file number for local ethical committee Arnhem-Nijmegen region: 2021-13214). All participants have provided informed e-consent for the use of their data to answer the research questions of this study. Data were pseudonymized using an encrypted code to which only treating health care professionals had access.

## Results

### Demographics

Demographics for participants in DS2 are presented in Table 1. Demographics for participants in DS1 were limited considering data of participants and centers was anonymized. The median age in DS1 was 26 (IQR 16-37) years, age was missing for 9 participants. The sex distribution in DS1 was 51% (307/601) male, 44% (263/601) female, and 5% (31/601) unspecified. The median number of participants per center in DS1 was 127 (IQR 82-153).

**Table 1:** Demographics of people with cystic fibrosis in dataset 2.

People with CF^a^ (n=81)			Values
Male sex, n (%)			40 (49.4)
Age at enrolment on the RMP^b^, year, median (IQR)			27 (12-40.5)
**Age distribution at enrolment on the RMP, n (%)**			
	<12 years	17 (21)	
	12 to <18 years	9 (11)	
	18 to <30 years	19 (24)	
	≥30 years	36 (44)	
Age range (years) at enrolment on the RMP, range			5 to 59
**Hospital, n (%)**			
	Radboud University Medical Center	14 (17)	
	HagaZiekenhuis	16 (20)	
	Maastricht University Medical Center+	26 (32)	
	University Medical Center Groningen	12 (15)	
	Amsterdam University Medical Center	13 (16)	
**CFTR** ^c^ **genotype, n (%)**			
	F508del homozygous	49 (61)	
	F508del heterozygous	27 (33)	
	Other	5 (6)	
**Highest FEV_1_** ^d^ **in 2020, (n=77)**			
	*z* score FEV_1_, median (IQR)	–2.0 (–4.4 to –0.9)	
	FEV_1_ percentage predicted, median (IQR)	75.1 (58.2-89.6)	

^a^CF: cystic fibrosis.

^b^RMP: remote monitoring program.

^c^CFTR: cystic fibrosis transmembrane conductance regulator.

^d^FEV_1_: forced expiratory volume in one second.

### Accuracy

**Figure 1:**
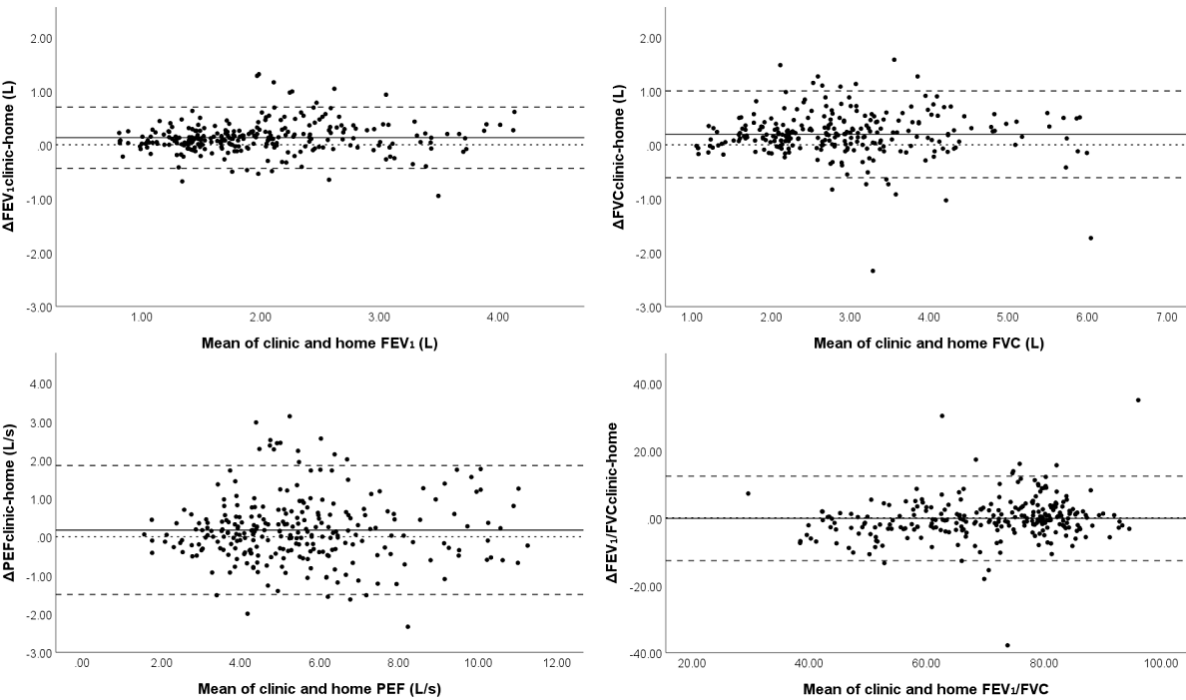
Bland-Altman plots of clinic measurements (n=263) compared with nearest neighbor home measurement (±7 days) for spirometry outcomes. From upper left to lower right: FEV1, FVC, PEF, and FEV1/FVC. X-axis: mean of clinic and home measurement; y-axis: clinic minus home measurement. N=263. Dotted line: reference line of equality (ie, Δclinic-home=0); Solid line: average bias (ie, average Δclinic-home); Dashed line: 95% limits of agreement. FEV1: forced expiratory volume in 1 second; FVC: forced vital capacity; PEF: peak expiratory flow.

In total, 86 and 263 pairs of home and clinic spirometry were made for same-day and NN measurements, respectively. For same-day pairs, 49 unique individuals had a median of 1 (IQR 1-2) pair. For NN pairs, 69 unique individuals had a median of 3 (IQR 1-5) pairs. Bland-Altman plots showed no visual heteroscedasticity (ie, variability of the differences remained similar as the magnitude of the mean value increased [14]) for NN pairs (Figure 1) nor same-day pairs (Multimedia Appendix 1) for all outcomes. Therefore, mean differences should be expressed as absolutes rather than percentages [14]. On average, home FEV_1_ and FVC were significantly lower than clinic spirometry for both NN and same-day pairs (Table 2). Limits of agreements were slightly narrower for same-day FEV_1_ and FVC pairs compared with NN pairs but remained similar for PEF and FEV_1_/FVC (Table 2).

**Figure 2:**
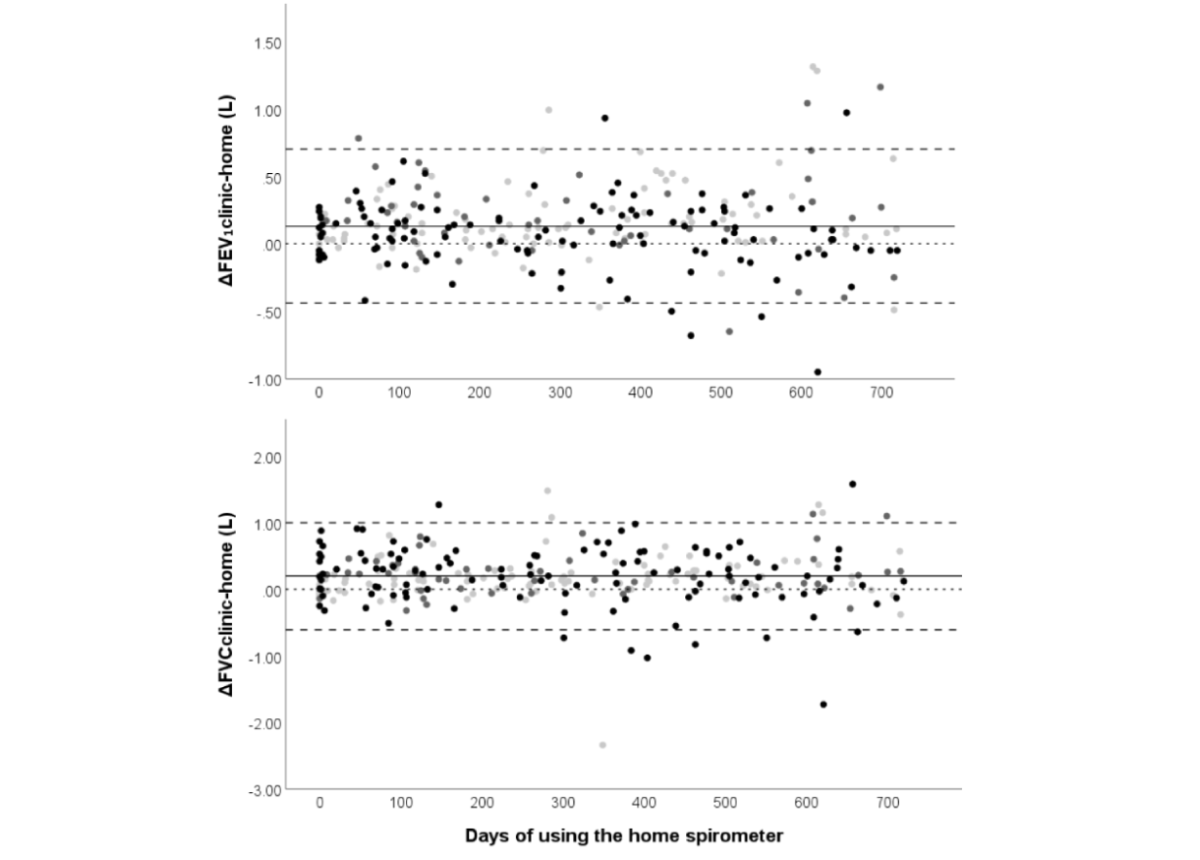
Bland-Altman plots of the absolute difference between clinic spirometry and nearest neighbor home spirometry (±7 days) by the number of days after the individual participants’ start of the home spirometer. Upper plot: FEV1; Lower plot: FVC. The color of points indicates age categories at the time of measurement. Light gray: 6 to 12 years old; gray 12 to 18 years old; and black: 18 years or older. N=263. Dotted line: reference line of equality (ie, Δclinic-home=0); Solid line: average bias (ie average Δclinic-home); Dashed line: 95% limits of agreement. FEV1: forced expiratory volume in 1 second; FVC: forced vital capacity.

To assess the effect of repeated measures, separate Bland-Altman plots using the participants’ individual contributions to the total spirometry pairs were performed. Within the contribution plots, individual participants were well mixed with each other, and variation in ΔFEV_1clinic-home_ and ΔFVC_clinic-home_ remained similar meaning an aggregate Bland-Altman analysis was deemed appropriate (Multimedia Appendix 2). In separate plots for age categories, adults had more ΔFEV_1clinic-home_ and ΔFVC_clinic-home_ pairs below the line of equality (FEV_1_: 34.8%, 40/115; FVC 28.7%, 33/115) than children (FEV_1_: 15.2%, 15/99; FVC: 23.2%, 23/99) and adolescents (FEV_1_: 24.5%, 12/49; FVC: 20.4%, 10/49) meaning clinic spirometry was more frequently lower than home spirometry in adults than in children or teenagers (Multimedia Appendix 3). Figure 2 shows Bland-Altman plots for NN FEV_1_ and FVC comparing ΔFEV_1clinic-home_ and ΔFVC_clinic-home_ by the number of days of experience users had with the portable spirometer.

One-way ANOVA showed a significant difference between age categories and NN ΔFEV_1clinic-home_ (*F*_2,121.66_=5.61, *P*<.01) but not for NN ΔFVC_clinic-home_, same day ΔFVC_clinic-home_, and same day ΔFEV_1clinic-home_. Gabriel post hoc test showed that adults (≥18 years, n=115) had on average lower ΔFEV_1clinic-home_ than children (6-12 years, n=99) (Δmeans=0.11 L, 95% CI –0.20 to –0.01, *P*=.02) and teenagers (12-18 years, n=49; Δmeans=0.14, 95% CI –0.25 to –0.02, *P*=.01). There was no statistically significant difference between children and teenagers (*P*=.93).

**Table 2:** Accuracy outcomes for forced expiratory volume in 1 second, forced vital capacity, and peak expiratory flow for paired home and clinic spirometry measurements on the same day and within 7 days from each other (nearest neighbor).

			Mean bias (sample mean Δclinic-home)		Estimated 95% CI of population mean Δclinic-home		95% limits of agreement of mean Δclinic-home
**Forced expiratory volume in 1 second**							
	Same day (n=86)	0.10 L^a^		0.05 to 0.14^a^		–0.32 to 0.52	
	Nearest neighbor (n=263)	0.13 L^a^		0.10 to 0.17^a^		–0.44 to 0.70	
**Forced vital capacity**							
	Same day (n=86)	0.22 L^a^		0.15 to 0.30^a^		–0.48 to 0.92	
	Nearest neighbor (n=263)	0.20 L^a^		0.14 to 0.25^a^		–0.61 to 1.00	
**Peak expiratory flow**							
	Same day (n=86)	0.12 L		–0.06 to 0.31		–1.60 to 1.85	
	Nearest neighbor (n=263)	0.17 L^a^		0.07 to 0.28^a^		–1.50 to 1.85	
**Forced expiratory volume in 1 second/forced vital capacity (Tiffeneau-index)**							
	Same day (n=86)	–0.53		–1.84 to 0.78		–12.49 to 11.43	
	Nearest neighbor (n=263)	–0.15		–0.92 to 0.63		–12.70 to 12.41	

^a^95% CI of mean absolute difference is outside the line of equality in Bland-Altman plots.

### Reproducibility

In total, there were 2669 unique measurement days of 311 people with CF with 2 or more home spirometry measurements on the same day. The median of unique measurement days per participant was 4 (IQR 2–10; range 1-265). Overall, 87.3% (2331/2669) of unique measurement days met repeatability criteria (ie, the difference between the largest and second-largest FEV_1_ or FVC ≤0.150L) for FEV_1_ compared with 74.3% (1985/2669) for FVC. In a sensitivity analysis, only the last measurement of every individual was included: 81% (252/311) of unique measurement days met repeatability criteria for FEV_1_ compared with 62.4% (194/311) for FVC. Median intraperson repeatability rates were 100% (IQR 89%-100%) for FEV_1_ and 72% (IQR 33%-96%) for FVC. The median intraperson CV was 2.80% (IQR 1.93%-4.69%) for FEV_1_ and 3.92% (IQR 2.45%-6.43%) for FVC.

Repeatability rates and CV between age groups are summarized in Table 3. Kruskal-Wallis with pairwise comparison demonstrated that both for FEV_1_ and FVC, CV was significantly affected by age categories (FEV_1_: *H*(2)=140.94, *P*<.001; FVC: *H*(2)=64.28, *P*<.001). For FEV_1_, pairwise comparisons with adjusted *P* values showed that there were small statistically significant differences in CV among all age categories: children versus adults (*P*<.001, *r*=0.24), teenagers versus adults (*P*<.001, *r*=0.16), and children versus teenagers (*P*<.001, *r*=0.10). For FVC, pairwise comparisons also revealed small significant differences in CV between children and adults (*P*<.001; *r*=0.16) and teenagers and adults (*P*<.001; *r*=0.10). There were no significant differences between children and teenagers (*P*=.06; *r*=0.07).

**Table 3:** Reproducibility outcomes within subgroups.

			Acceptable repeatability				Coefficient of variation		
			FEV_1_^a^, n (%)		FVC^b^, n (%)		FEV_1_ (%), median (IQR)		FVC (%), median (IQR)
**Age categories**									
	Children (6-12 years, n=492)	424 (86.2)		392 (79.7)		3.49 (1.88-6.33)		3.74 (1.82-6.20)	
	Teenagers (12-18 years, n=504)	386 (76.6)		348 (69)		2.82 (1.34-5.78)		3.02 (1.56-5.67)	
	Adults (18 years or older, n=1583)	1437 (90.8)		1174 (74.2)		1.91 (1.05-3.46)		2.41 (1.27-4.53)	
**Days of using the home spirometer**									
	0-100 days (n=676)	574 (84.9)		468 (69.2)		2.80 (1.36-5.29)		3.31 (1.60-6.30)	
	300-400 days (n=311)	283 (91)		247 (79.4)		2.13 (1.18-3.79)		2.36 (1.40-4.54)	
	600-700 days (n=178)	155 (87.1)		131 (73.6)		2.23 (1.18-4.01)		2.53 (1.32-5.23)	

^a^FEV_1_: forced expiratory volume in 1 second.

^b^FVC: forced vital capacity.

### Quality Grading

Overall, 47.7% (2435/5104) home spirometry tests received grade E for both FEV_1_ and FVC because only one measurement was performed. For tests with more than one measurement, FEV_1_ grades were A (43.8%, 1168/2669), B (43.6%, 1163/2269), C (4.5%, 119/2669), D, (2.1%, 56/2669), and E (6.1%, 163/2669). FVC grades were A (28%, 748/2669), B (46.3%, 1237/2669), C (7.6%, 203/2669), D (4.9%, 132/2669), and E (13.1%, 349/2669). For individuals’ final tests only, FEV_1_ grades were A (27.7%, 86/311), B (53.4%, 166/311), C (6.8%, 21/311), D (2.9%, 9/311); and E (9.3%, 29/311). Final FVC grades were A (15.4%, 48/311), B (46.9%, 146/311), C (8.7%, 27/311), D (6.4%, 20/311), and E (22.5%, 70/311).

### Responsiveness to Treatment

**Figure 3:**
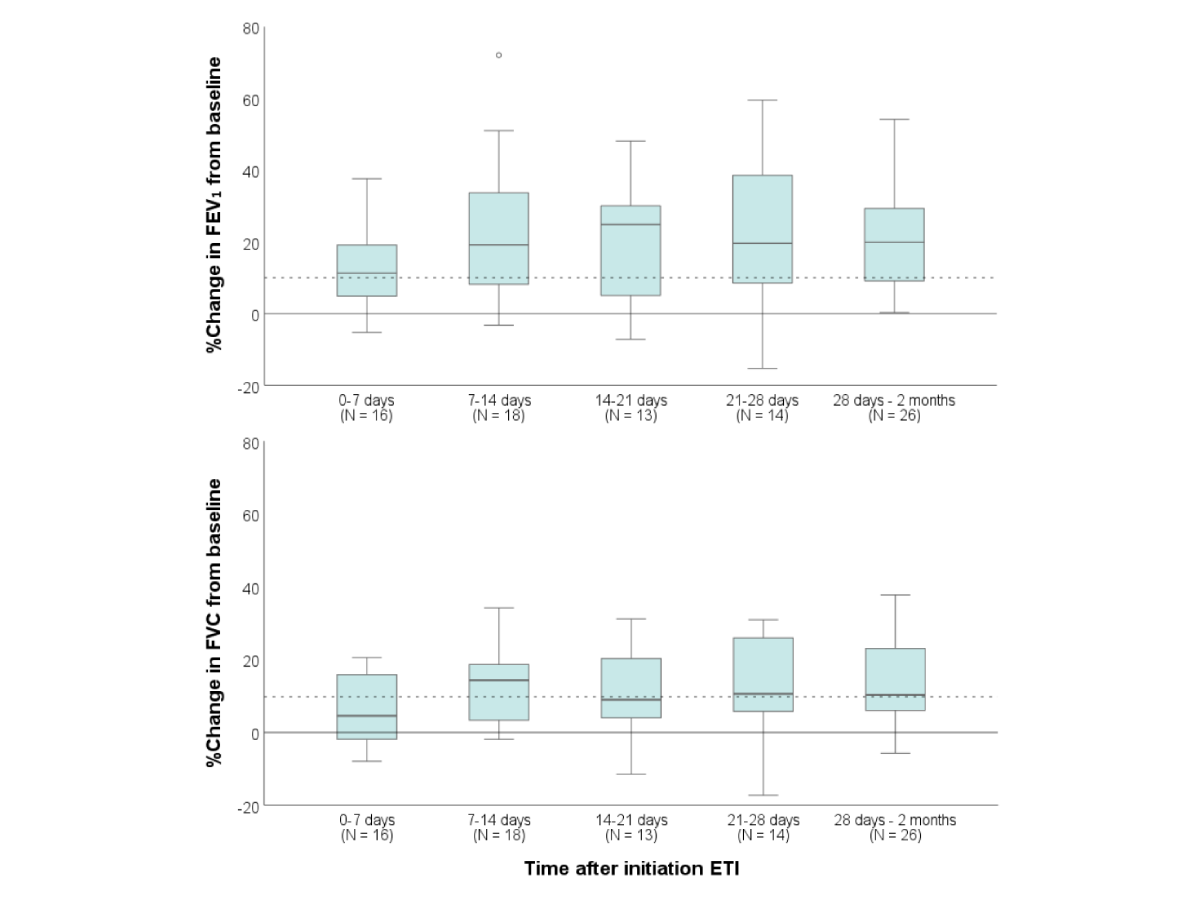
Boxplots visualizing the absolute percentage change from baseline for FEV1 (upper panel) and FVC (lower panel) after initiation of ETI. Dashed line: minimal clinically important difference for FEV1 and FVC (10% change). The total amount of participants is 33. Baseline FEV1 and FVC were calculated as the average FEV1 and FVC within the first 2 months before initiation of ETI. ETI: elexacaftor/tezacaftor/ivacaftor; FEV1: forced expiratory volume in 1 second; FVC: forced vital capacity.

A total of 73 people with CF in DS2 had started ETI during the study period, of which 33 had at least one baseline home and follow-up home spirometry measurement. Median absolute percentage changes from baseline after initiation of ETI were as follows: FEV_1_: 11.3% (IQR 4.2%-19.2%), FVC: 4.5% (IQR –2.2%-15.8%) after 0-7 days; FEV_1_: 19.2% (IQR 7.9%-34.8%), FVC: 14.3% (IQR 3.3%-19.1%) after 7-14 days; and thereafter FVC and FEV_1_ remained relatively stable (Figure 3). After 2 weeks, 66.7% (12/18) of users who had measured their lung function improved more than 10% in home FEV_1_.

## Discussion

### Principal Findings

This study aimed to assess the accuracy, reproducibility, and responsiveness to the treatment of home spirometry in long-term CF care. Our findings show that home spirometry outcomes are significantly lower than clinic spirometry in both children and adults with broad 95% limits of agreement that surpass repeatability criteria. For ΔFVC_clinic-home_ but not ΔFEV_1clinic-home_, the mean difference also surpassed the 150mL limit of repeatability [15,16]. For all assessed outcomes, there was no heteroscedasticity in our population meaning that the mean bias and limits of agreement are similar for low and high absolute values, which has important implications for people with CF with lower lung volumes. The reproducibility of home spirometry was high, especially for FEV_1_, but the quality of tests could be improved by increasing the amount of maneuvers during home spirometry measurements. Finally, home spirometry was responsive to ETI treatment and showed clinically significant changes in lung function in 2 thirds of participants.

### Comparison to Previous Work

Our findings closely resemble those by Edmondson et al [7] who presented a similar mean bias of 0.14 L with limits of agreement of ±0.40 L for unsupervised home FEV_1_ in children using a Vitalograph portable spirometer without training effects after 6 months. They also found a significant systemic underread of the portable spirometers as tested with calibrated syringes [7]. Bell et al [10] found that virtual supervision of home spirometry with Air-Next or Spirohome spirometers was not different from unsupervised FEV_1_ home measurements. Taken together, multiple sources of bias and combinations could be underlying lower home spirometry outcomes. First, portable spirometers could have a systematic underread bias but it is unclear whether this is present in all different models, devices, and applications, and how this is affected by a lack of daily calibration such as performed in the clinic. Future research should assess whether periodical calibration of home spirometers during outpatient visits is possible and whether it improves long-term accuracy. Second, a lack of supervision could still cause lower home spirometry results for some people with CF as studies that compare unsupervised and supervised measurements are constrained by Hawthorne effects and selection biases. Finally, our findings show increased variability of home spirometry after multiple years of use. People with CF, especially children, have previously reported that home spirometry was more difficult than clinic spirometry and long-term use might introduce imprudence [3]. Therefore, repeated training might be necessary to prevent further deviation rather than improving underread biases, but this should be subject to further investigation.

We found that children and teenagers more often had somewhat lower at-home FEV_1_ and FVC than adults compared to those measured by in-clinic spirometry. Only one study has previously compared children with adults and reported that adults and teenagers had less variable and more reliable home spirometry than children [11]. Other studies have also argued that children perform better than teenagers, which has been ascribed to more supervision from parents [6,7]. However, these findings were not replicated in our study. We previously found that children have more difficulty with home spirometry as the technique is different and the mouthpiece is relatively large for them [3] Moreover, children and teenagers appeared less patient and motivated for spirometry within their private setting than adults, especially when parents press them to perform measurements at home [3]. Therefore, impatience and imprudence, but also issues with technique or the large mouthpiece offer alternative explanations for less reliable results in children compared to adults.

Overall, the intratest reproducibility of home spirometry was good for FEV_1_ with statistical but clinically irrelevant differences between age categories. The experience did not seem to impact intratest reproducibility outcomes. FVC had less favorable intratest reproducibility and quality grades than FEV_1_, possibly because it requires a more challenging maneuver. For FEV_1_, quality grades in our study were similar to those found earlier in adults, but quality grades for home FVC in CF have not yet been reported [10]. The most important improvement in quality would be better instruction to include more maneuvers in measurements as most measurements only included one maneuver.

Finally, we found that within 2 weeks after initiation of ETI, a median increase of 20% in FEV_1_ and 15% in FVC from baseline could be quantified with home spirometry in a subgroup of users. Unfortunately, we were unable to calculate *z* scores or percentage predictive values for these home measurements in this study which made statistical analyses and comparison with literature difficult [17,18]. Nevertheless, 2 thirds of users improved more than 10% in FEV_1_, which is a clinically relevant change in lung function [2,15,16]. Considering that home spirometers consistently underestimate clinic spirometry rather than overestimate, a clinically significant improvement (ie, >10%) in short-term home spirometry is still highly relevant and will likely be even larger in the clinic. This may make home spirometers better suited to confirm short-term treatment responses remotely than to rule out treatment responses. The use of home spirometry in treatment responses should be subject to further study.

### Strengths and Limitations

To our knowledge, this is the largest study on accuracy, reproducibility, and responsiveness of home spirometry that was performed within a multicenter and long-term setting and also included participants from all age categories. Therefore, our findings will likely more closely resemble actual home spirometry accuracy as there was less selection bias or Hawthorne effects compared to prospective studies. This study had 2 important limitations. First, the use of anonymous data meant there was no information on pulmonary exacerbations or the use of therapies such as antibiotic courses or short-acting beta-agonist therapies which might cause differences between home and clinic measurements. We aimed to mitigate these effects by analyzing both NN and same-day accuracy. As the same day and NN findings closely resembled each other, we are confident that the effect of this was inconsequential in our study. Second, a small group of individuals had missing data on age which could not be corrected due to the use of anonymous data and these individuals had to be excluded from age group comparisons.

### Clinical Implications

Home spirometry, especially home FEV_1_, can be performed acceptably at home but is not equal to clinic spirometry. FVC home measurements might pose more challenges as a remotely measured outcome considering the average bias surpassed repeatability criteria, 95% limits of agreement were broad, and acceptability grades were lower. Consequently, home spirometry can offer an alternative to clinic spirometry in the follow-up of people with CF or to provide point-of-care lung function data remotely, but home spirometry should not be used to make diagnoses or to detect subtle changes in pulmonary function.

Home spirometry can be used to confirm that a patient’s lung function is stable when outcomes are comparable to recent clinic spirometry. However, the context of a measurement as well as the deviation from a personal baseline might be more clinically relevant than the actual home spirometry values [3] Therefore, education of patients about the possibility of lower values and the need for home spirometry measurements with more than one maneuver on regular intervals is essential. This will prevent concerns or disappointments of patients and enable the assessment of deterioration or improvement from personal baselines. Finally, home spirometers should regularly be taken into the clinic during routine outpatient visits to assess individual deviation from clinic measurements and to evaluate the technique by professional pulmonary function technicians.

### Conclusions

Home spirometry offers a feasible opportunity to collect lung function outcomes remotely but is not equal to clinic spirometry. Home spirometry can be offered to people with CF who are familiar with pulmonary function tests as a tool for remote follow-up during periods of stability, for remote point-of-care lung function outcomes during deterioration, or to enable and evaluate home treatments. Considering the consistent underread, home spirometry might be well suited to rule out deterioration when values remain stable but should be interpreted with caution when values are lower than expected. Future research should focus on the role of training and user familiarity with portable spirometry devices on the long-term reliability of home spirometry and on the role of home spirometry in assessing treatment responses in CF and other chronic respiratory diseases.

## Appendix

Multimedia Appendix 1 Bland-Altman plots for same day clinic and home spirometry pairs. From upper left to lower right: FEV1, FVC, PEF, and FEV1/FVC.

Multimedia Appendix 2 Bland-Altman plots for nearest neighbor FEV1 and FVC with individual clinic and home spirometry pairs categorized by the participants’ individual contributions to the total amount of spirometry pairs in the dataset. Blue: the participant contributed less than one percent of the overall amount of spirometry pairs; Green: the participant contributed between one and three percent of spirometry pairs; Red: the participant contributed more than three percent of spirometry pairs. Panel 1: FEV1; panel 2: FVC.

Multimedia Appendix 3 Bland-Altman plots for nearest neighbor FEV1 with individual clinic and home spirometry pairs.

## Abbreviations

ATS/ERS: American Thoracic Society/European Respiratory Society
CF: cystic fibrosis
CFTR: cystic fibrosis transmembrane conductance regulator
CV: coefficient of variation
DS1: dataset 1
DS2: dataset 2
ETI: elexacaftor/tezacaftor/ivacaftor
FEV1: forced expiratory volume in one second
FVC: forced vital capacity
NN: nearest neighbor
PEF: peak expiratory flow
RMP: remote monitoring program

## Data Availability

The datasets generated during and/or analyzed during this study are available from the corresponding author on reasonable request.

## References

[1] Shteinberg M, Haq IJ, Polineni D, Davies JC (2021). Cystic fibrosis. The Lancet.

[2] Castellani C, Duff AJA, Bell SC, Heijerman HGM, Munck A, Ratjen F, Sermet-Gaudelus I, Southern KW, Barben J, Flume PA, Hodková P, Kashirskaya N, Kirszenbaum MN, Madge S, Oxley H, Plant B, Schwarzenberg SJ, Smyth AR, Taccetti G, Wagner TO, Wolfe SP, Drevinek P (2018). ECFS best practice guidelines: the 2018 revision. J Cyst Fibros.

[3] Oppelaar MC, Emond Y, Bannier MAGE, Reijers MHE, van der Vaart H, van der Meer R, Altenburg J, Conemans L, Rottier BL, Nuijsink M, van den Wijngaart LS, Merkus PJFM, Heinen M, Roukema J (2024). Potential, pitfalls, and future directions for remote monitoring of chronic respiratory diseases: multicenter mixed methods study in routine cystic fibrosis care. J Med Internet Res.

[4] Thornton CS, Magaret AS, Carmody LA, Kalikin LM, Simon RH, LiPuma JJ, Caverly LJ (2024). Quantifying variation in home spirometry in people with cystic fibrosis during baseline health, and associations with clinical outcomes. J Cyst Fibros.

[5] Paynter A, Khan U, Heltshe SL, Goss CH, Lechtzin N, Hamblett NM (2022). A comparison of clinic and home spirometry as longtudinal outcomes in cystic fibrosis. J Cyst Fibros.

[6] Gerzon F, Jöbsis Q, Bannier M, Winkens B, Dompeling E (2020). Discrepancy between lung function measurements at home and in the hospital in children with asthma and CF. J Clin Med.

[7] Edmondson C, Westrupp N, Short C, Seddon P, Olden C, Wallis C, Brodlie M, Baxter F, McCormick J, MacFarlane S, Brooker R, Connon M, Ghayyda S, Blaikie L, Thursfield R, Brown L, Price A, Fleischer E, Hughes D, Donnelly C, Rosenthal M, Wallenburg J, Brownlee K, Alton EWFW, Bush A, Davies JC (2023). Unsupervised home spirometry is not equivalent to supervised clinic spirometry in children and young people with cystic fibrosis: results from the CLIMB-CF study. Pediatr Pulmonol.

[8] Davis J, Ryan M, Marchetti P, Dahlberg SE, Greenberg J, Bacon C, Kaur R, Scalia S, Sawicki GS (2022). Real-world feasibility of short-term, unsupervised home spirometry in CF. Pediatr Pulmonol.

[9] Fettes E, Riley M, Brotherston S, Doughty C, Griffiths B, Laverty A, Aurora P (2022). "You're on mute!" Does pediatric CF home spirometry require physiologist supervision?. Pediatr Pulmonol.

[10] Bell JM, Sivam S, Dentice RL, Dwyer TJ, Jo HE, Lau EM, Munoz PA, Nolan SA, Taylor NA, Visser SK, Yozghatlian VA, Wong KK (2022). Quality of home spirometry performance amongst adults with cystic fibrosis. J Cyst Fibros.

[11] Beaufils F, Enaud R, Gallode F, Boucher G, Macey J, Berger P, Fayon M, Bui S (2023). Adherence, reliability, and variability of home spirometry telemonitoring in cystic fibrosis. Front Pediatr.

[12] Medical International Research (2018). Spirobank Smart. User Manual Rev 2.4.

[13] Miller MR, Hankinson J, Brusasco V, Burgos F, Casaburi R, Coates A, Crapo R, Enright P, van der Grinten CPM, Gustafsson P, Jensen R, Johnson DC, MacIntyre N, McKay R, Navajas D, Pedersen OF, Pellegrino R, Viegi G, Wanger J, ATS/ERS Task Force (2005). Standardisation of spirometry. Eur Respir J.

[14] Giavarina D (2015). Understanding bland altman analysis. Biochem Med (Zagreb).

[15] Graham BL, Steenbruggen I, Miller MR, Barjaktarevic IZ, Cooper BG, Hall GL, Hallstrand TS, Kaminsky DA, McCarthy K, McCormack MC, Oropez CE, Rosenfeld M, Stanojevic S, Swanney MP, Thompson BR (2019). Standardization of spirometry 2019 update. An Official American thoracic society and European respiratory society technical statement. Am J Respir Crit Care Med.

[16] Culver BH, Graham BL, Coates AL, Wanger J, Berry CE, Clarke PK, Hallstrand TS, Hankinson JL, Kaminsky DA, MacIntyre NR, McCormack MC, Rosenfeld M, Stanojevic S, Weiner DJ (2017). Recommendations for a standardized pulmonary function report. An Official American thoracic society technical statement. Am J Respir Crit Care Med.

[17] Sutharsan S, McKone EF, Downey DG, Duckers J, MacGregor G, Tullis E, Van Braeckel E, Wainwright CE, Watson D, Ahluwalia N, Bruinsma BG, Harris C, Lam AP, Lou Y, Moskowitz SM, Tian S, Yuan J, Waltz D, Mall MA, Aurora P, Verhulst S, Watson D, Lorenz M, Roehmel J, Gleiber W, Naehrig S, Stehling F, Sutharsan S, van Koningsbruggen-Rietschel S, Fischer R, Downey D, Haworth C, Duckers J, Legg J, Barry P, Thursfield R, Doe SJ, Hilliard T, MacGregor G, Nash EF, Withers NJ, Peckham D, Barr HL, Lee T, Gray R, Vermeulen F, Van Braeckel E, Vanderhelst E, Robinson PJ, Wainwright CE, Smith DJ, Mulrennan SA, Clements BS, Wark P (2022). Efficacy and safety of elexacaftor plus tezacaftor plus ivacaftor versus tezacaftor plus ivacaftor in people with cystic fibrosis homozygous for F508del-CFTR: a 24-week, multicentre, randomised, double-blind, active-controlled, phase 3b trial. The Lancet Respiratory Medicine.

[18] Mall MA, Brugha R, Gartner S, Legg J, Moeller A, Mondejar-Lopez P, Prais D, Pressler T, Ratjen F, Reix P, Robinson PD, Selvadurai H, Stehling F, Ahluwalia N, Arteaga-Solis E, Bruinsma BG, Jennings M, Moskowitz SM, Noel S, Tian S, Weinstock TG, Wu P, Wainwright CE, Davies JC (2022). Efficacy and safety of elexacaftor/tezacaftor/ivacaftor in children 6 through 11 years of age with cystic fibrosis heterozygous for and a Minimal Function Mutation: A Phase 3b, Randomized, Placebo-controlled Study. Am J Respir Crit Care Med.

