# Supplementary material for: Accuracy, Reproducibility, and Responsiveness to Treatment of Home Spirometry in Cystic Fibrosis: Multicenter, Retrospective, Observational Study
Source: J Med Internet Res. 2024 Dec 3;26:e60892. doi: 10.2196/60892 (PMC11653036; doi:10.2196/60892)
Supplement: Multimedia Appendix 1 [file jmir_v26i1e60892_app1.docx]

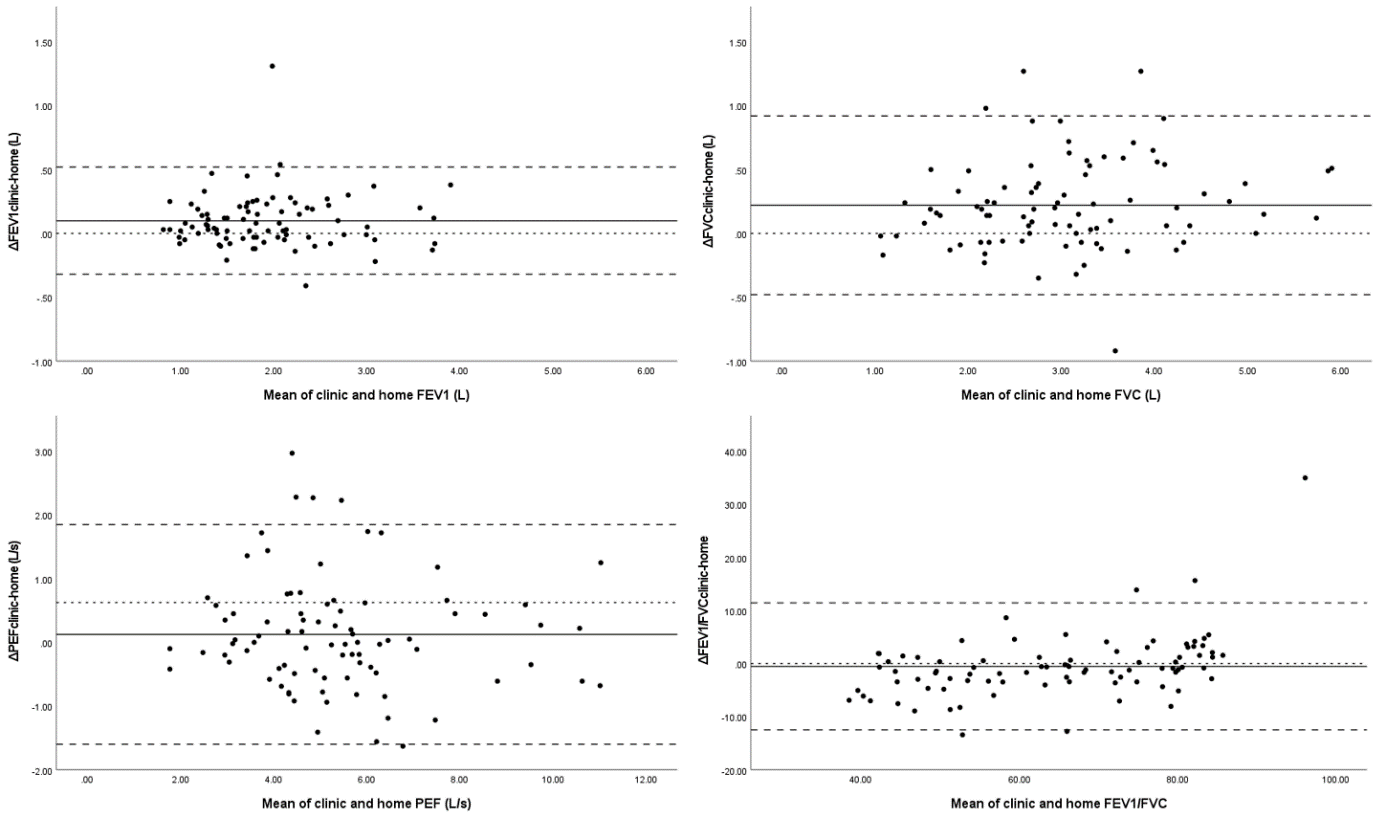


**Figure S1:** Bland-Altman plots for same day clinic and home spirometry pairs. From upper left to lower right: FEV1, FVC, PEF, and FEV1/FVC.
