# Supplementary material for: Accuracy, Reproducibility, and Responsiveness to Treatment of Home Spirometry in Cystic Fibrosis: Multicenter, Retrospective, Observational Study
Source: J Med Internet Res. 2024 Dec 3;26:e60892. doi: 10.2196/60892 (PMC11653036; doi:10.2196/60892)
Supplement: Multimedia Appendix 2 [file jmir_v26i1e60892_app2.docx]

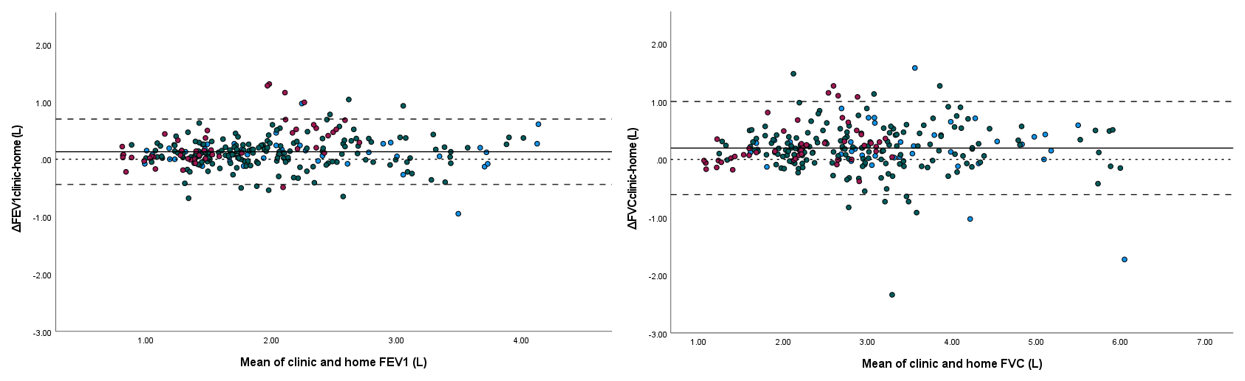


**Figure 2:** Bland-Altman plots for nearest neighbour FEV1 and FVC with individual clinic and home spirometry pairs categorized by the participants’ individual contributions to the total amount of spirometry pairs in the dataset. Blue : the participant contributed less than one percent of the overall amount of spirometry pairs; Green : the participant contributed between one and three percent of spirometry pairs; Red : the participant contributed more than three percent of spirometry pairs. Panel 1: FEV1; panel 2; FVC
