# Supplementary material for: Accuracy, Reproducibility, and Responsiveness to Treatment of Home Spirometry in Cystic Fibrosis: Multicenter, Retrospective, Observational Study
Source: J Med Internet Res. 2024 Dec 3;26:e60892. doi: 10.2196/60892 (PMC11653036; doi:10.2196/60892)
Supplement: Multimedia Appendix 3 [file jmir_v26i1e60892_app3.docx]

***
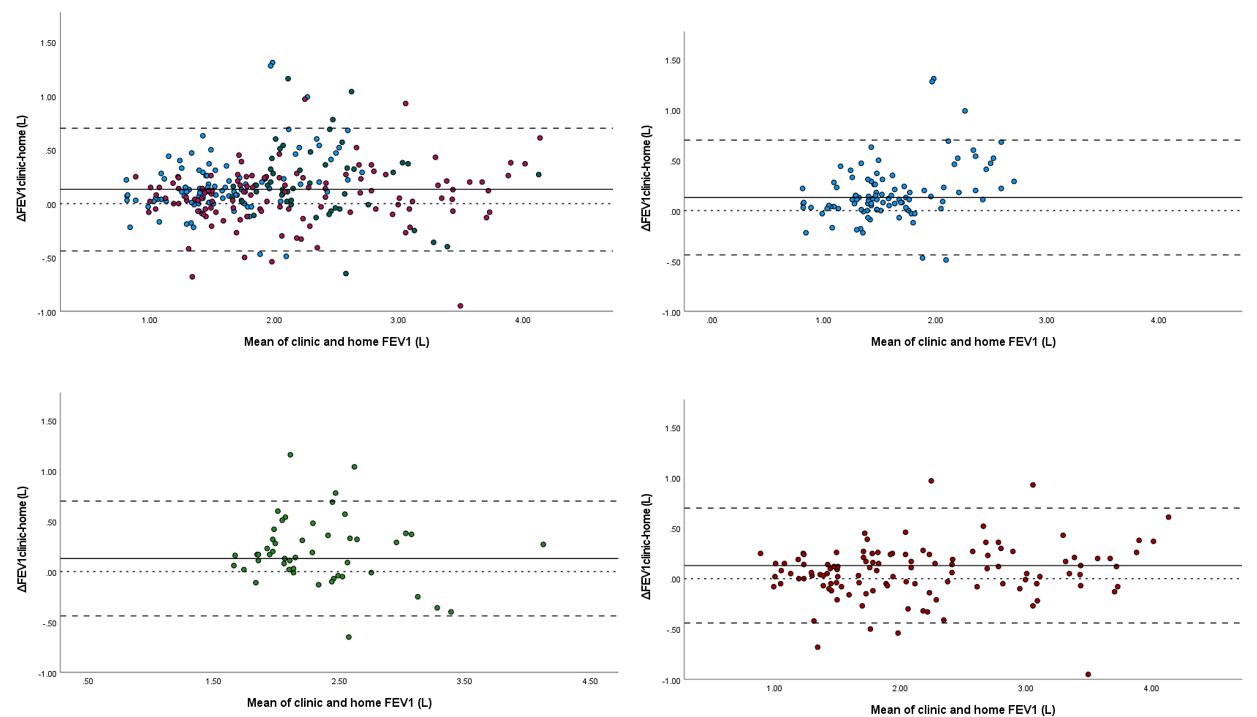
***

*
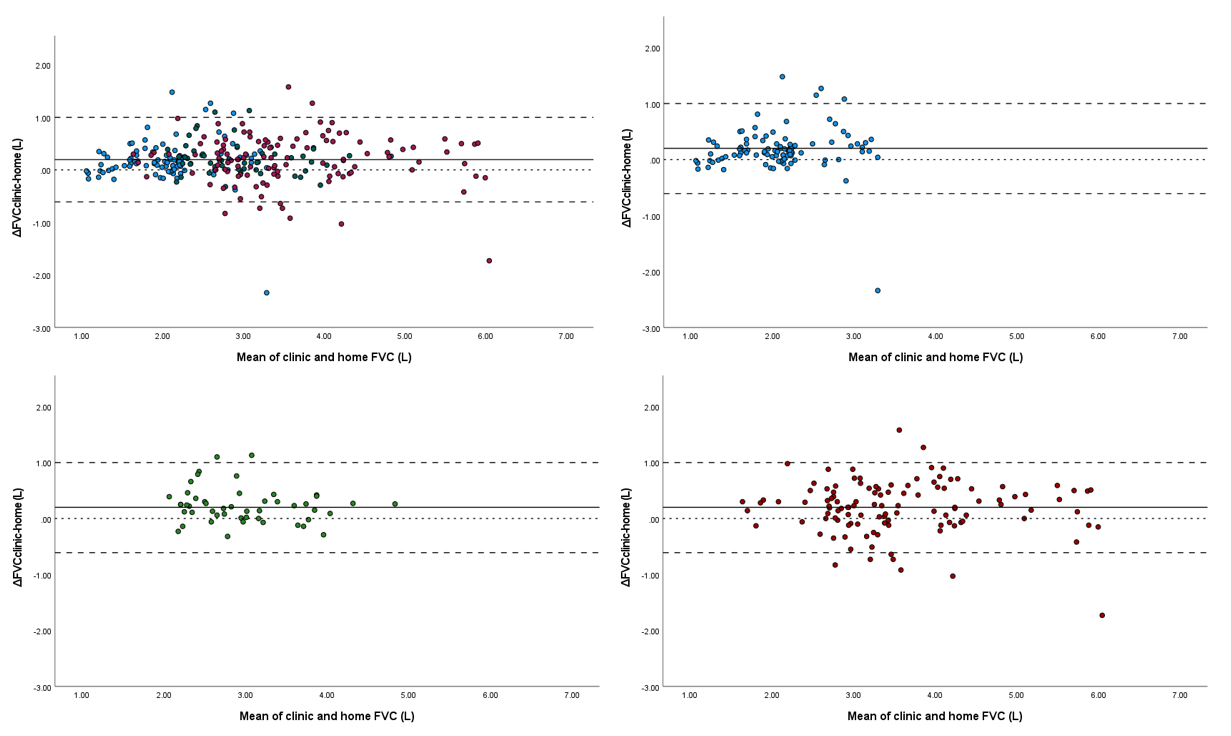
***Figure 3a:** Bland-Altman plots for nearest neighbour FEV1 with individual clinic and home spirometry pairs categorized by the participants’ age category at the time of the clinic measurement. Blue : 6 to 12 years of age; Green : 12 to 18 years of age; Red : 18 years or older. From upper left to right: age categories combined, 6 to 12 years old, 12 to 18 years old; 18 years old or olde

**Figure 3b:** Bland-Altman plots for nearest neighbour FVC with individual clinic and home spirometry pairs categorized by the participants’ age category at the time of the clinic measurement. Blue : 6 to 12 years of age; Green : 12 to 18 years of age; Red : 18 years or older. From upper left to right: age categories combined, 6 to 12 years old, 12 to 18 years old; 18 years old or older

*.*

***Supplemental***
